# Activation of ClO_2_ by Nanoscale Zero-Valent Iron for Efficient Soil Polycyclic Aromatic Hydrocarbon Degradation: New Insight into the Relative Contribution of Fe(IV) and Hydroxyl Radicals

**DOI:** 10.3390/toxics13010036

**Published:** 2025-01-05

**Authors:** Xiaojun Hu, Xiaorong Xing, Fan Zhang, Bingzhi Li, Senlin Chen, Bo Wang, Jiaolong Qin, Jie Miao

**Affiliations:** 1 School of Chemical and Environmental Engineering, Shanghai Institute of Technology, Shanghai 201418, China; hu-xj@sit.edu.cn (X.H.); 18303582575@163.com (X.X.); 17836203056@163.com (F.Z.); libingzhi2000@zju.edu.cn (B.L.); 15716394694@163.com (S.C.); wangb@sit.edu.cn (B.W.); 2 School of Environmental Science and Engineering, Nanjing Tech University, Nanjing 211816, China

**Keywords:** Fe(IV), hydroxyl radicals, polycyclic aromatic hydrocarbons, chlorine dioxide, nanoscale zero-valent iron

## Abstract

Recently, the activation of chlorine dioxide (ClO_2_) by metal(oxide) for soil remediation has gained notable attention. However, the related activation mechanisms are still not clear. Herein, the variation of iron species and ClO_2_, the generated reactive oxygen species, and the toxicity of the degradation intermediates were explored and evaluated with nanoscale zero-valent iron (nFe^0^) being employed to activate ClO_2_ for soil polycyclic aromatic hydrocarbon (PAH) removal. With an optimized ClO_2_/nFe^0^ molar ratio of 15:1 and a soil/water ratio of 3:1, the degradation efficiency of phenanthrene improved 12% in comparison with that of a ClO_2_-alone system. The presence of nFe^0^ significantly promoted ClO_2_ consumption (improved 85.4%) but restrained ClO_2_^−^ generation (reduced 22.5%). The surface Fe(II) and soluble Fe(II) in the ClO_2_/nFe^0^ system was 2.0-fold and 2.8-fold that in the nFe^0^ system after 2 min. Electron paramagnetic resonance analysis, along with quenching experiments, revealed that Fe(IV), HOCl, and •OH dominated phenanthrene degradation in a ClO_2_/nFe^0^ system, with oxidation contributions, respectively, of 34.3%, 52.8% and 12.9%. The degradation intermediates of PAHs in the ClO_2_/nFe^0^ system had lower estimated toxicity than those of the ClO_2_ system. The lettuces grown in ClO_2_/nFe^0^-treated soil displayed better results in bioassay indexes than those grown in ClO_2_-treated soil. This study offers new perspectives for the remediation of organic-pollutant-contaminated soil by using metal-activated ClO_2_ technology.

## 1. Introduction

Polycyclic aromatic hydrocarbons (PAHs), many of which are human carcinogenic, teratogenic, and ecotoxic, are generated when organic matter and fossil fuels incompletely combust [1]. PAHs pose serious threats to human health and ecological systems by way of amplification effects in the food chain [2,3]. Sixteen parent PAHs have been grouped into the priority pollutants category by the U.S. Environmental Protection Agency [4]. Due to their hydrophobic nature and persistence, PAHs can be found ubiquitously in the environment, with soil being their ultimate sink [5,6]. The removal of PAHs from soil is a challenge, because PAHs are strongly adsorbed into soil organic matter and are encapsulated in soil minerals [6,7]. Thus, developing effective methods to remediate PAH-contaminated soil is urgently necessary.

As a one-electron oxidant (E_0_ = 0.936 V), chlorine dioxide (ClO_2_) has the advantage of having less pH dependence and lower dissolved organic form disinfection byproduct formation potential in comparison with free chlorine. ClO_2_-based advanced oxidant processes have been widely applied in water and wastewater treatment [8,9,10,11]. Peng et al. [8] reported reactive species (ClO•, Cl•, •OH, and ozone) generated when ClO_2_ was activated by UV radiation in the UVA range. Su et al. [11] reported that active ClO_2_ radicals and photo-induced •OH radicals were produced in a combined ClO_2_-photocatalysis system, which significantly increased the degradation rate of norfloxacin. Gaseous ClO_2_ has been proven to have an antimicrobial effect on pathogenic bacteria and has been used as a substitute soil fumigant instead of methyl bromide [12,13]. Our previous work showed that ClO_2_ was an effective oxidizing agent for PAHs in soil [14]. The mechanism involves ClO_2_ attacking the atoms of PAHs which have the strongest electron-donating ability. However, developing ClO_2_-based technologies for soil remediation is restricted by lengthy reaction times, low treatment efficiency, high selectivity for organic pollutants, and high costs with huge ClO_2_ dosages [15,16]. Recently, the activation of ClO_2_ by metal or metal oxide has gained a large amount of interest. Shi et al. [17] employed carbon–MnO_2_ to catalyze ClO_2_ for the enhanced removal of o-chlorophenol from wastewater. Ma et al. [15] reported that montmorillonite-supported Fe_3_O_4_-CuO exhibited excellent catalytic activity in the ClO_2_ catalytic oxidation process for anthracene (ANT) degradation in soil. Wang et al. [16] reported that the degradation efficiency of 2-sec-butyl-4,6-dinitrophenol was significantly improved when ClO_2_ was heterogeneously catalyzed by Al_2_O_3_. Our previous work [18] showed that divalent manganese ions could activate ClO_2_ for PAH removal from contaminated industrial soil, with the principal active species HOCl and •OH. This research showed that the activation of ClO_2_ by metal or metal oxide should alleviate the drawbacks seen when ClO_2_ is used alone. However, the present metal catalysts (Mn- or Cu-based catalysts) pose heavy metal pollution risks to soil. Thus, much more environmentally friendly and effective materials need to be explored for the activation of ClO_2_ for soil PAH degradation.

With the advantages of being low-cost, environmentally harmless, and commercially available, nanoscale zero-valent iron (nFe^0^) has been broadly applied to reduce contaminants in water or soil by itself [19,20,21,22,23], or along with the activation of persulfate [24,25]. Micro-scale Fe^0^ has been reported to be an excellent activator for ClO_2_ activation for p-nitrophenol removal in aqueous solutions [26]. It is known that ClO_2_ inevitably forms chlorite (ClO_2_^−^) by reacting with pollutant components through a one-electron transfer process (Equation (1)) [27]. Terhalle et al. [28] reported that each consumed ClO_2_ could generate 62% relative ClO_2_^−^ and 42% relative hypochlorous acid (HOCl) when ClO_2_ reacts with organic compounds (e.g., phenol). Aguilar et al. reported on the generation of •OH when using soluble Fe(II) and HOCl due to a Fenton-like reaction (Equation (2)) [29,30]. In addition, Fe(IV) was reported to be generated on nFe^0^ surfaces when using ClO_2_^−^ as the oxidant (Equation (3)) [31]. Significantly, soluble Fe(II) species, which can be easily generated during nFe^0^ oxidation, have been shown to effectively react with ClO_2_^−^ (Equation (4)) [32,33]. Therefore, nFe^0^ is a promising activator for the activation of ClO_2_, but the activation mechanisms, the variation of iron species, and the generated reactive oxygen species are unclear. In addition, nFe^0^ could act like a fertilizer by promoting plant photosynthesis and iron uptake [20,34,35]. However, no known studies have been conducted describing the degradation routes of PAHs and evaluating the biological toxicity of PAH-contaminated soil treated by a ClO_2_/nFe^0^ system. Thus, a study concerning the use of nFe^0^ to activate ClO_2_ for the removal of PAHs in soil is worth conducting.

(1) $$C_{6}H_{6}O+{\mathrm{ClO}}_{2}\to C_{6}H_{4}O_{2}+\;\mathrm{HOCl}\;+\;{\mathrm{ClO}}_{2}^{-}$$

(2) $$\mathrm{HOCl}+{\mathrm{Fe}}^{2+}\to {\mathrm{Fe}}^{3+}+\cdot \mathrm{OH}\;+\;{\mathrm{Cl}}^{-}$$

(3) $$\equiv {\mathrm{Fe}(\mathrm{II})+\mathrm{ClO}}_{2}^{-}\to \equiv \mathrm{Fe}\left(\mathrm{IV}\right)=O\;+\;{\mathrm{ClO}}^{-}$$

(4) $${\mathrm{ClO}}_{2}^{-}+4\;{\mathrm{Fe}}^{2+}+4\;H^{+}\to {\mathrm{Cl}}^{-}+4\;{\mathrm{Fe}}^{3+}+2\;H_{2}O$$

Herein, the performance of nFe^0^-activated ClO_2_ for PAH removal from soil was investigated. Phenanthrene (PHE) was chosen as a typical PAH to prepare spiked soil, because it belongs to the sixteen parent PAHs which have been grouped into the priority pollutants category by the U.S. Environmental Protection Agency [4]. The main purposes were as follows: (1) to optimize the reaction condition for PAH removal in a ClO_2_/nFe^0^ system by investigating the effect of the water/soil ratio, ClO_2_ dosage, ClO_2_/nFe^0^ mole ratio and reaction pHs; (2) to clarify the ClO_2_ activation mechanisms in the ClO_2_/nFe^0^ system by analyzing iron species, exploring ClO_2_ variations, and detecting the reactive oxygen species through ERR analysis and quenching experiments; (3) to evaluate the toxicity of the degradation intermediates of PAHs in the ClO_2_/nFe^0^ system by using quantitative structure–activity relationship prediction technology and detecting lettuces’ growth in ClO_2_/nFe^0^-treated soil.

## 2. Materials and Methods

### 2.1. Chemicals

Chemicals are listed in detail in Text S1. The PHE-spiked soil samples were prepared as follows: grassland soil samples (5~10 cm) were collected from Fengxian campus in the Shanghai Institute of Technology (Shanghai, China) and dried. Table S1 lists the physicochemical characteristics of the soil.

### 2.2. Preparation of PHE-Spiked Soil, ClO_2_ and nFe^0^

The total iron in soil was analyzed using a previously reported method [36]. The soil was grounded and sieved through a 65-mesh screen. The PHE (10 mg) in 100 mL of n-hexane/dichloromethane (1: 1 *v*/*v*) solvent was mixed with the soil (100 g) in a large beaker by magnetic stirring. The PHE-spiked soil was aged in the dark to volatilize the solvent, for at least two weeks, and was used for further experiments.

ClO_2_ was prepared by the sodium chlorite–sulfuric acid method according to previous research [37]. In brief, 250 mL sulfuric acid solution (0.1 M) was added to 500 mL sodium chlorite solution (0.4 M) by using a peristaltic pump. The generated ClO_2_ gas was taken out of the reaction flask by N_2_-purging and purified through a saturated NaClO_2_ solution. The prepared ClO_2_ solution was put into a 4 °C refrigerator. The exact concentration of the ClO_2_ solution was detected before each use [38].

nFe^0^ was prepared by using NaBH_4_ to reduce FeSO_4_ into Fe^0^ in a liquid phase [39]. Deionized water (100 mL) was added into a 250 mL three-necked flask with N_2_ was purged for 0.5 h. Then, FeSO_4_·7H_2_O (8.00 mg) was put into the flask and stirred at 250 rpm. Sodium borohydride (3.96 g) was dissolved in water (100 mL) and added to flask by peristaltic pump at 4.50 mL/min. The suspension was kept purging N_2_ for 2 h, and then it was filtered. The precipitates were dried in a vacuum oven for 4 h. Then, the dried nFe^0^ was collected in a centrifugal tube for further use. The surface morphology and species changes of nFe^0^ were detected by scanning electron microscopy with energy dispersive spectroscopy (SEM-EDS, ZEISS, Oberkochen, Germany), X-Ray Diffractometer (XRD, D8 Advance davinci, Bruker, Ettlingen, Germany) and an XPS instrument (Thermo Scientific Escalab 250Xi, Waltham, MA, USA). The surface area was analyzed with the Brunauer–Emmett–Teller (BET) nitrogen adsorption technique (ASAP 2010 M+C, Micrometitics Inc., Norcross, GA, USA).

### 2.3. Batch Degradation Experiments

The batch experiments concerning soil PHE degradation were conducted in 20 mL brown bottles with lids at a constant agitation of 250 (±5) rpm at 25 (±1) °C, avoiding light. The desired PHE-containing soil (3 g) was added to the bottle, followed by proportions of water (i.e., 3, 6, 9, 12, 15 g), ClO_2_ (i.e., 40, 80,160 mM/kg), and nFe^0^ (i.e., 0.25, 0.33, 0.50, 1.00, 2.50 g/kg). The effect of varied pHs (3.0~9.0) on PAH degradation in the nFe^0^/ClO_2_ system and the ClO_2_ system was studied, since the soil pH usually ranged from about 6.0 to 8.5 [40]. The pH adjustment of the soil suspension was conducted using 0.1 M of HCl or 0.1 M of NaOH. The different experimental conditions in each batch are summarized in Table S2. The pH variations are presented in Table S3. The extraction procedures for residual PAHs followed the USEPA test method 3550B [41,42]. When reaching the predetermined time, the bottles were immediately put into a −20 °C refrigerator to terminate the reaction for 12 h. After freeze-drying, 5 mL of n-hexane–dichloromethane mixed solvent with a volume ratio of 1: 1 was added to each bottle. The bottle was put on a horizontal oscillator at 250 rpm for 1 h at 25 °C. To extract the residual PAHs in the soil, the capped bottle was ultrasonicated at 400 W for 1 h. After that, the extracting liquid was filtrated through a 0.22 μm PTFE filter membrane and the filtrate was collected for analysis. The recovery rate of this extraction method was around 88%. A GC-MS (Shimadzu, Kyoto, Japan, QP-2010 Ultra) with an SH-Rxi-5Sil-MS column (30 m × 0.25 mm × 0.25 μm, 0.25 μm) was used for the detection of PAHs and intermediates. The carrier gas was helium, and the flow rate was 1.0 mL/min. The injection port was kept at 280.0 °C, and the heating procedure was maintained at 60 °C for 1 min, then increased to 200 °C at 10 °C/min, and then increased to 300 °C at 5 °C/min for 8 min. The MS spectra reading, with an electron impact source at 70 eV, an ion source temperature of 230 °C, and analysis were performed in the SIM mode. When identifying the degradation products, the column temperature programmed for degradation product identification was as follows: starting at 50 °C, to be maintained for 1 min, then warmed from 50 °C to 300 °C at a speed of 5 °C/min, and then maintained at a constant temperature of 300 °C for 5 min, after which identification and analysis of PHA intermediates was performed in SCAN mode. 

### 2.4. Detection of Iron Species and ClO_2_ Variations

A UV-vis spectrometry device (UV-5500, Metash, Shanghai, China) was employed to analysis iron species at 510 nm [43]. To put it simply, the total Fe(II), including soluble Fe (II) [Fe(II)_sol_] and surface Fe(II) [Fe(II)_surf_], was measured by adding 1,10-phenanthroline before filtration, while Fe(II)_sol_ was determined by adding 1,10-phenanthroline after filtration. The concentration of Fe (II)_surf_ was obtained by calculating the differentials between total Fe (II) and Fe(II)_sol_. The concentration of ClO_2_ was detected by using UV-vis method at 359 nm and ClO_2_^-^ was detected at 260 nm after removing ClO_2_ with N_2_-blowing [44].

### 2.5. Identification of Active Oxidant Species

The active oxidant species in the reaction system were identified by using 5,5-dimethyl-1-pyrroline-oxide (DMPO) as a spin trapper with a Bruker micro-ESR device (Standard V2.0, Bruker, Ettlingen, Germany). The resonance frequency was 9.704 G, and the scan time and scan number were 15.5 s and 32, respectively [45]. Methyl phenyl sulfoxide (PMSO), Isopropyl alcohol (IPA), and glycine (Gly) were used as scavengers for Fe(IV), hydroxyl radical (•OH), and hypochlorous acid (HOCl), respectively [14]. The quenching experiments were conducted in PHE-polluted water (1 mg/L) to exclude interferences by soil constitution [11,28,46]. The scavengers were added before oxidant addition, with a scavenger/ClO_2_ molar ratio of 5:1. As iron minerals in soil mainly existed in the form of hematite (Fe_2_O_3_) and goethite (FeOOH) [47,48], the reactive oxygen species in the ClO_2_/Fe_2_O_3_ and ClO_2_/FeOOH systems were detected to evaluate interferences by the original iron species in the soil.

According to previous studies, Fe(IV) oxidizes PMSO to produce methyl phenyl sulfone (PMSO_2_) (Equation (5)), while free radicals can oxidize PMSO to produce hydroxylation products (Equation (6)) [49,50,51]. Therefore, PMSO and PMSO_2_ were, respectively, detected, to examine the contribution of Fe(IV) in the reaction system by using HPLC (Shimadzu, Japan) with an SPD-M20A detector. Text S2 listed the detailed parameters of HPLC, and in Figure S1 the chromatograms of PMSO and PMSO_2_ are presented.

(5) $$C_{7}H_{8}OS+\mathrm{Fe}\left(Ⅳ\right)=O\;\to C_{7}H_{8}O_{2}S+\mathrm{Fe}(Ⅱ)\;$$

(6) $$C_{7}H_{8}OS+\cdot \mathrm{OH}\;\to C_{6}H_{6}O_{2}S+\cdot {\mathrm{CH}}_{3}$$

### 2.6. Biological Toxicity Evaluation

The growth of lettuce in PHE-spiked soil treated by ClO_2_/nFe^0^ or ClO_2_ alone were explored, respectively. Lettuce seeds were evenly put on filter paper in a Petri dish for seed germination, and the filter paper was moistened with deionized water. The lettuce seedlings were obtained by incubating the Petri dish at room temperature for 2 days. ClO_2_/nFe^0^-treated soil or ClO_2_-treated soil were used to study lettuce growth, respectively. A total of 100 g of treated soil was placed into each of three pots, with one lettuce seedling planted in each pot. The pots were incubated under light for 16 h and in the dark for 8 h, at room temperature [52]. Water (2 mL) was poured into each pot every day. The plant length, width, and dry weight of lettuce leaves were measured at the selected time. The pots filled with un-contaminated grassland soil were set as the experiment control (CK).

### 2.7. Data Analysis

Each set of treatments was repeated three times. Data analysis and plotting were performed using origin 2017 (OriginLab, Northampton, MA, USA). The significance of differences between parallel experiments were assessed by using the minimum significance difference postmortem test and univariate with SPSS 24.0 software (SPSS, Inc., Chicago, IL, USA) at a significance level of *p* < 0.05.

## 3. Results and Discussion

### 3.1. Optimization of the Reaction Conditions for PHE Degradation in ClO_2_/nFe^0^ System

**Figure 1:**
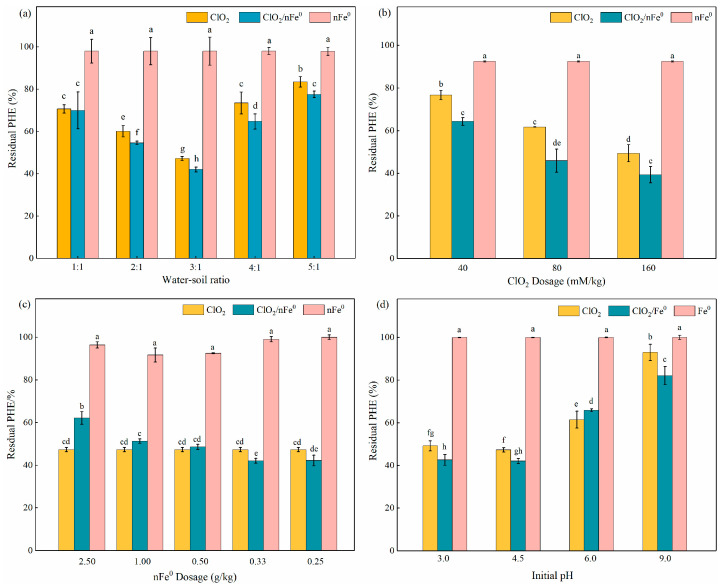
Effect of water/soil ratio (**a**), chlorine dioxide dosage (**b**), nFe^0^ dosage (**c**) and initial pH (**d**) on the removal of PHE in soil in different reaction systems. Experimental conditions: [PHE]= 100 mg/kg; T = 25 °C; reaction time = 12 h. The different lowercase letters above the bars indicate groups with significant differences (*p* < 0.05).

Measurement of the effect of the water/soil ratio ranging from 1:1 to 5:1 on PHE degradation was conducted in the ClO_2_/nFe^0^ system and the ClO_2_ system, respectively (Figure 1a). As the water/soil ratio increases from 1:1 to 5:1, the degradation of PHE firstly increased by 27.9% (from 30.0% to 57.9%), then decreased by 35.5% (from 57.9% to 22.4%), with the highest degradation of 57.9% occurring at a water/soil ratio of 3:1 in the ClO_2_/nFe^0^ system. The reason could be that the increasing of soil moisture to a proper proportion (e.g., 3:1) made part of the pollutants on the soil surface quickly disperse into the aqueous solution. Thus, the contact between pollutants and reactive oxidant species enhanced greatly, with more pollutants desorbed from soil to water at a higher water/soil ratio [53,54]. However, when the water/soil ratio surpassed 3:1, the reactive oxidant species may be consumed by dissolved organic matter before they react with the target pollutants, which was in keeping with previous research [55,56]. In addition, Figure 1a also showed that the removal efficiency of PHE in a ClO_2_/nFe^0^ system was higher (5.2~8.7%) than that in the ClO_2_-alone system, at different water/soil ratios (1:1 except). Based on the above results, a water/soil ratio of 3:1 was recommended for the following experiments.

Pre-experiments showed that the cumulative degradation of PAHs may be negligible, since the PAH residuals were around 93.0–98.0% after 12 h of treatment in the nFe^0^-alone system. Figure 1b showed the degradation of PHE in soil with varied dosages of ClO_2_ and a fixed nFe^0^ dosage (0.33 mg/kg). The residual PAHs significantly decreased from 64.3% to 39.4% as the ClO_2_ dosage increased from 40 to 160 mM/kg. When the dosage of ClO_2_ was 40 mM/kg, the stoichiometric efficiency (or the utilization efficiency), defined as the amount of PHE (g) transformed per gram of ClO_2_ consumed, was 4.3. The utilization efficiency decreased from 3.3 to 1.8 as the ClO_2_ dosage increased from 80 to 160 mM/kg. The ideal dosage of ClO_2_ was selected as 80 mM/kg soil, based on the results for degradation efficiency and utilization efficiency.

Different dosages of nFe^0^ were tested to investigate its effect on the degradation of PHE in the presence of 80 mM/kg of ClO_2_ (Figure 1c). When the dosage of nFe^0^ was less than 0.33 g/kg, the degradation of PHE in ClO_2_/nFe^0^ system was apparently higher than that in ClO_2_-alone system. When the nFe^0^ dosage exceeded 0.33 g/kg, the degradation of PHE decreased. The presence of excess nFe^0^ could result in the generation of excess Fe(II) species (Equation (7)), which may act as a scavenger to ClO_2_ and ClO_2_^−^ via parasitic reactions (Equations (4) and (7)) [32,57,58]. The reduced ClO_2_ and ClO_2_^−^ further decreased the generation of reactive oxygen species. Thus, the degradation of PHE decreased with the excess in nFe^0^. Overall, based on the degradation performance of PHE, the best dosage of nFe^0^ was determined to be 0.33 g/kg with the molar ratio of ClO_2_/nFe^0^ of 15:1.

(7) $${\mathrm{ClO}}_{2}+5\;{\mathrm{Fe}}^{2+}+4\;H^{+}\to {\mathrm{Cl}}^{-}+5\;{\mathrm{Fe}}^{3+}+2\;H_{2}O$$

The effect of different initial pHs (3.0, 4.5, 6.0, and 9.0) on the degradation of PAHs was shown in Figure 1d. As the pH increased from 3.0 to 9.0, the degradation efficiency of PHE reduced from 57.5% to 18.0% in the ClO_2_/nFe^0^ system, and from 50.8% to 7.0% in the ClO_2_ system. This indicated that acidic conditions favored PHE degradation in the two systems. The reason could be that the increase in pH improved the precipitation tendency for the release of Fe(II) and reduced the generation of reactive oxide species [59,60,61]. Figure 1d also indicated that the presence of nFe^0^ in ClO_2_ system increased the degradation efficiency of PHE by approximately 6.5%, 5.2%, and 10.9% at a pH of 3.0, 4.5, and 9.0, respectively. However, when the pH was nearly neutral (pH = 6.0), the removal of PHE in the ClO_2_/nFe^0^ system (34.1%) was slightly lower (4.4%) than that in the ClO_2_-alone system (38.5%). This could be attributed to the reactive oxidant species in the ClO_2_/nFe^0^ system, which were much more sensitive to the decline of pH in comparison with those in the ClO_2_ system. Nevertheless, future experiments are required to determine the changes in reactive oxidant species with varied pHs. The above results indicated that the ClO_2_/nFe^0^ system showed superiority over ClO_2_ for PHE removal at varied pHs. In comparison to other advanced oxidant process-based strategies (Table S4), ClO_2_/nFe^0^, for soil PAHs degradation, has a major advantage in high-oxidant-utilization efficiency, with a small oxidant/PAH ratio (*w*/*w*).

### 3.2. Characterization of nFe^0^

**Figure 2:**
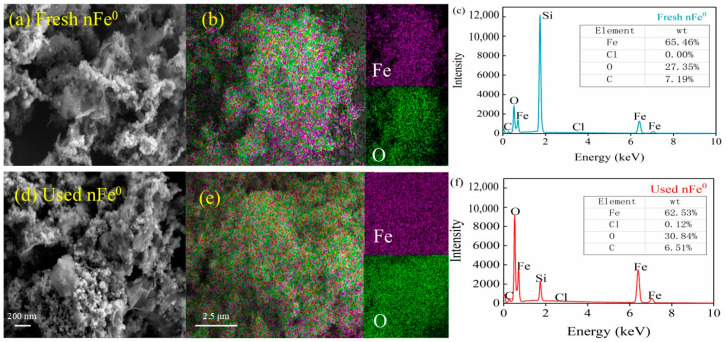
SEM-EDS analysis. SEM image of fresh nFe^0^ (**a**) and used nFe^0^ (**d**); EDS elemental mapping of fresh nFe^0^ (**b**) and used nFe^0^ (**e**); and the EDS spectrum of fresh nFe^0^ (**c**) and used nFe^0^ (**f**). The inset table shows the elemental composition.

**Figure 3:**
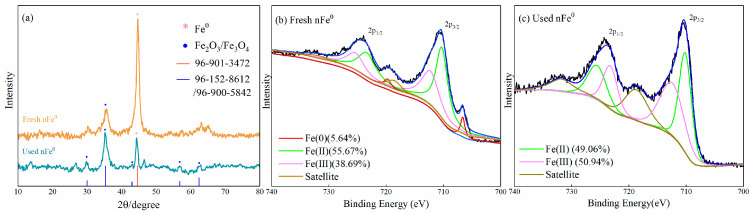
XRD spectra (**a**) and high-resolution XPS spectra of Fe 2p spectra for fresh nFe^0^ (**b**) and used nFe^0^ (**c**).

The fresh and used nFe^0^ were characterized by SEM-EDS (Figure 2). The nFe^0^ was a spherical particle with a diameter of approximately 25 nm and a BET surface of 73.46 m^2^/g. It remained spherical after oxidization by ClO_2_. The surface contains 33.7% Fe and 49.1% O. After the reaction, the composition of Fe decreased to 31.2% and O increased to 53.7%. In addition, Cl (0.1%) was also detected after the reaction. The variance in elemental constituents indicated oxidation of Fe^0^ by ClO_2_ (Figure 2f). Figure 3a showed the XRD patterns of original and used nFe^0^. Two Fe^0^ characteristic peaks could be found both in fresh and used Fe^0^ patterns at 2θ values of 44.13° and 64.48°, respectively, indicating that the Fe^0^ was the main structural composition of the fresh nFe^0^ (COD2021 standard card 96-901-3472) [62]. The diffraction peaks (30.05°, 35.39°, 43.01°, 56.88°, and 62.46°) of Fe_3_O_4_ (maghemite, COD2021 standard card 96-900-5842) or Fe_2_O_3_ (magnetite, COD2021 standard card 96-152-8612) appeared in the fresh nFe^0^, indicating the partial oxidation of fresh nFe^0^ [63,64]. After the reaction, the characteristic peaks of Fe^0^ were weaker, but the characteristic peaks of magnetite/maghemite were stronger than those of the fresh nFe^0^. This indicated that Fe^0^ was consumed and iron oxide was generated in the ClO_2_/nFe^0^ oxidation system.

The surface elemental composition and chemical state of fresh and used nFe^0^ were characterized by XPS (Figure 3b,c). According to the Fe 2p spectrum, 706.78, 710.25, and 712.61 eV were, respectively, attributed to Fe(0), Fe(Ⅱ), and Fe(Ⅲ) [65]. After participating in degradation, the characteristic peak of Fe(0) disappeared. However, the composition of Fe(Ⅱ) increased from 42.22% to 53.35%, and that of Fe(Ⅲ) increased from 44.61% to 46.65%. This indicated electron transfer in the nFe^0^, and the formation of iron oxide on the surface. According to the O 1s spectrum (Figure S2), the peaks at 529.5 and 531.0 eV corresponded to the lattice oxygen (O_latt_), Fe-O, and surface-absorbed oxygen (O_abs_) [66]. After participating in the reaction, the surface lattice oxygen (O_latt_) Fe-O content of nFe^0^ increased from 46.72% to 53.36%, indicating the oxidation of Fe^0^, while the O_abs_ gradually decreased from 53.28% to 46.64%, indicating that during the ClO_2_/nFe^0^ process, the formation of an oxide layer on the nFe^0^ surface occurred.

### 3.3. Reactive Oxygen Species in ClO_2_/nFe^0^ System

**Figure 4:**
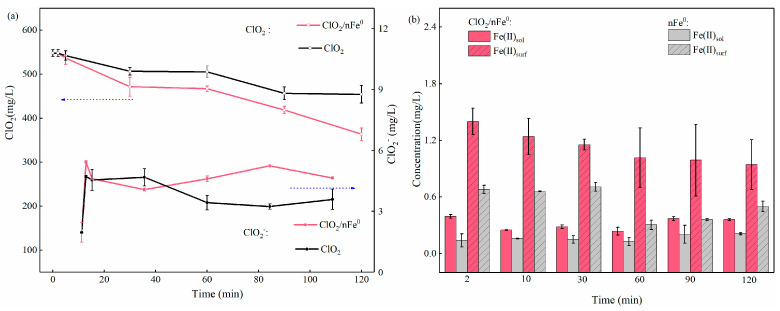
(**a**) The concentration of ClO_2_ and ClO_2_^−^ in ClO_2_/nFe^0^ and ClO_2_ system. (**b**) The concentration of Fe(II)_sol_ and Fe(II)_surf_ in ClO_2_/nFe^0^ or nFe^0^ systems. Experimental conditions: [PHE] = 1 mg/L; [ClO_2_] = 548 mg/L; [nFe^0^] = 100 mg/L; T = 25 °C; pH = 4.5.

As a powerful one-electron oxidant, ClO_2_ could obtain an electron from the pollutant and be turned into ClO_2_^−^ (Equation (1)) [27]. To explore the intrinsic reaction, the concentrations of ClO_2_ and ClO_2_^−^ (Figure 4a) were detected during ClO_2_ activation by nFe^0^. The ClO_2_ concentration in the ClO_2_/nFe^0^ system is lower than that in the ClO_2_ system during the reaction, indicating that much more ClO_2_ was consumed in the presence of nFe^0^. For example, the consumed ClO_2_ in the ClO_2_/nFe^0^ system was 11.50 and 76.40 mg/L in the first 5 min and 30 min, but only 5.50 and 41.20 mg/L in the ClO_2_-alone system, indicating that the presence of Fe^0^ made the ClO_2_ be almost doubly consumed. However, the generated ClO_2_^−^ in the first 5 min was 2.63 mg/L in the ClO_2_/nFe^0^ system, which is comparable to that in the ClO_2_/nFe^0^ system (2.59 mg/L). At 30 min, the ClO_2_^−^ in ClO_2_/nFe^0^ (2.10 mg/L) was 0.77-fold that of the ClO_2_ system (2.71 mg/L), respectively. This indicated that the presence of nFe^0^ did not significantly contribute to the generation of ClO_2_^−^, but significantly promoted the consumption of ClO_2_. To further clarify the reaction between ClO_2_ and nFe^0^, Fe(II)_surf_ and Fe(II)_sol_ were analyzed during the reaction and shown in Figure 4b. Compared to the nFe^0^-alone system, the presence of ClO_2_ increased both Fe(II)_surf_ and Fe(II)_sol_. At 2 min and 10 min, the concentration of Fe(II)_surf_ in ClO_2_/nFe^0^ was 2.0-fold and 1.9-fold that in the nFe^0^ system, and the concentration of Fe(II)_sol_ in ClO_2_/nFe^0^ was 2.8-fold and 1.6-fold that in the nFe^0^ system. In addition, the Fe(II)_surf_ concentration was 3.5-fold and 4.0-fold higher than the Fe(II)_sol_ concentration at 2 min and 10 min in the ClO_2_/nFe^0^ system, indicating that Fe(II)_surf_ was the main iron species. Accordingly, the concentration of Fe(III)_surf_ and Fe(III)_sol_ in ClO_2_/nFe^0^ was 10-fold and 1.5-fold that in the nFe^0^ system at 10 min (Figure S3), respectively. The concentration of Fe(III)_surf_ was 5.3-fold higher than Fe(III)_sol_. The results also showed that nFe^0^ was mainly turned into Fe(II)_surf_ and Fe(III)_surf_ by the provision of electrons to ClO_2_, similar to other nFe^0^-based (e.g., nFe^0^/persulfate) advanced oxidant processes [34,67]

However, though a higher proportion of ClO_2_ was consumed in the ClO_2_/nFe^0^ system, the generated ClO_2_^−^ was not accordingly increased. Therefore, other active species should have been generated by electron transfer between ClO_2_ and nFe^0^. It has been reported that the Cl–O bond of ClO_2_^–^ could dissociate and react with electron-donating Fe(II) species on an nFe^0^ surface to generate Fe(IV) (Equation (3)) [31]. Thus, with the existence of ClO_2_^−^ and Fe(II)_surf_ on nFe^0^, Fe(IV) should be generated in the ClO_2_/nFe^0^ oxidation system.

**Figure 5:**
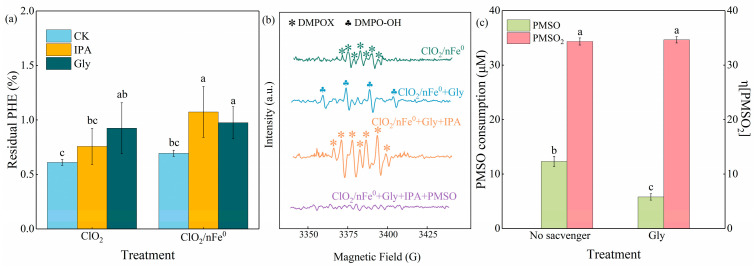
Quenching experiments and EPR spectra of ClO_2_/nFe^0^ system under DMPO capture agent. (**a**) Comparison of the quencher effects on the degradation of PHE in ClO_2_/nFe^0^ system and ClO_2_ system. (**b**) EPR spectra of DMPOX adducts with different scavengers in ClO_2_/nFe^0^ system. (**c**) Effect of Gly on the generation of PMSO and generation of PMSO_2_ at ClO_2_/nFe^0^ system. Experimental conditions: [PHE] = 1 mg/L; [ClO_2_] = 548 mg/L; [nFe^0^] = 100 mg/L; [Gly] = [IPA] = [PMSO] = 4 mM; [DMPO] = 100 mM; T = 25 °C; reaction time = 10 min. The different lowercase letters above the bars indicate groups with significant differences (*p* < 0.05).

The reactive oxygen species were further analyzed by EPR detection (Figure 5b). A seven-line signal attributed to the DMPOX adduct was observed both in the ClO_2_/nFe^0^ (Figure 5b) system and the ClO_2_-alone (Figure S4a) system, which might have been generated by the reaction between DMPO and reactive oxidant species (•OH, HOCl, and Fe(IV) species) [19,68]. No signal was detected in nFe^0^ (Figure S4a). The DMPOX signal in the ClO_2_/nFe^0^ system was much stronger in comparison with that in the ClO_2_-alone system, which indicated that more active substances were produced in the former system. When Gly was added as a specific scavenger of HOCl, the DMPOX signals instantly vanished in the ClO_2_-alone system (Figure S4a), suggesting that the primary reactive oxygen species responsible for DMPO oxidation was HOCl in the ClO_2_-alone system. However, after the scavenging of HOCl by Gly, the typical signals of DMPO-OH were observed in the ClO_2_/nFe^0^ system (Figure 5b), suggesting the existence of an •OH radical. With further addition of Gly and IPA to quench both •OH and HOCl, the DMPOX signals appeared again. Interestingly, when PMSO was further added as a specific scavenger for Fe(IV), the DMPOX signals disappeared immediately, suggesting Fe(IV) also exists in the ClO_2_/nFe^0^ system. The typical signals of DMPO-OH were not observed in the ClO_2_/Fe_2_O_3_ or the ClO_2_/FeOOH system (Figure S4b,c), let alone Fe(IV) species in the ClO_2_ system activated by iron minerals in soil. Thus, the iron minerals in soil cannot activate ClO_2_ to generate reactive oxidant species, and HOCl, •OH, and Fe(IV) were confirmed to exist only in the ClO_2_/nFe^0^ system (Scheme S1).

As HOCl and •OH were commonly detected when organic contaminants were oxidized by ClO_2_ or activated ClO_2_ [18,28], IPA and Gly were employed as scavengers for •OH and HOCl, respectively, in this study to explore the active species in a ClO_2_/nFe^0^ system (Figure 5). As shown in Figure 5a, compared to that in CK (without any scavenger), the addition of Gly markedly increased the residual PHE in a ClO_2_-alone system and the ClO_2_/nFe^0^ system. This indicated that HOCl is involved in the degradation of PHE in the two oxidation systems. The presence of IPA did not significantly affect the residual percentage of PHE in the ClO_2_-alone system, but resulted in a 55.0% increase in the residual PHE in the ClO_2_/nFe^0^ system. This showed that •OH contributed to PHE degradation in the ClO_2_/nFe^0^ system.

To further clarify the contribution of HOCl, •OH, and Fe(IV), PMSO was used as an Fe(IV) prober because Fe(IV) can selectively react with PMSO to generate PMSO_2_ [19]. As shown in Figure 5c, 12.3 μM of PMSO was consumed and 4.8 μM PMSO_2_ was generated in the ClO_2_/nFe^0^ system without any scavenger. Thus, the contribution percentage (η) of Fe(IV), which was defined as the molar ratio of generated PMSO_2_ to consumed PMSO, was 34.3% in ClO_2_/nFe^0^ system. Since the reaction rate of PMSO with •OH (*k*_PMSO, ·OH =_ 3.61 × 10^9^ M^−1^s^−1^) was matched to that of IPA with •OH (*k*_IPA, ·OH_ = 3 × 10^9^ M^−1^s^−1^), IPA was not a suitable scavenger for distinguishing the respective contribution of •OH and PMSO [51,69,70]. As a selective HOCl scavenger [28], Gly was added to the ClO_2_/nFe^0^ system to explore the oxidation contribution of HOCl. With the presence of Gly, the consumed PMSO reduced 52.8% (from 12.3 to 5.8 μM), indicating that HOCl contributed 52.8% to the oxidation system. The generated PMSO_2_ was 2.4 μM and the corresponding η (34.6%) was comparable to that in the oxidation system without any scavenger (34.3%). This showed that Gly hardly affects the function of Fe(IV). Since •OH, Fe(IV), and HOCl were the three main active oxygen species, the contribution of •OH should have been 12.9% (100%–34.3%–52.8%). Therefore, the respective oxidation contribution of Fe(IV), HOCl, and •OH were 34.3%, 52.8%, and 12.9% in the ClO_2_/nFe^0^ system. However, the actual oxidant efficiency of the three main active oxygen species for PHE removal is worthy of research in the future.

### 3.4. Degradation Intermediates and Ecotoxicity Prediction

**Figure 6:**
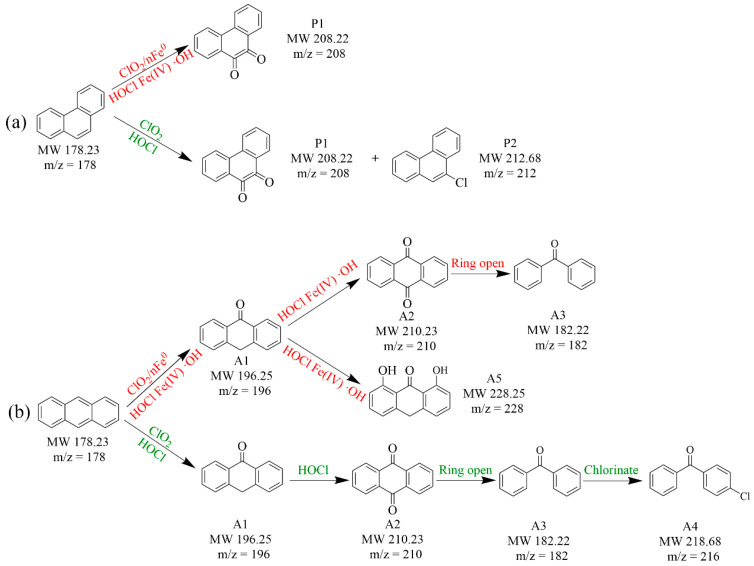
Proposed degradation pathways of PHE (**a**) and ANT (**b**).

**Figure 7:**
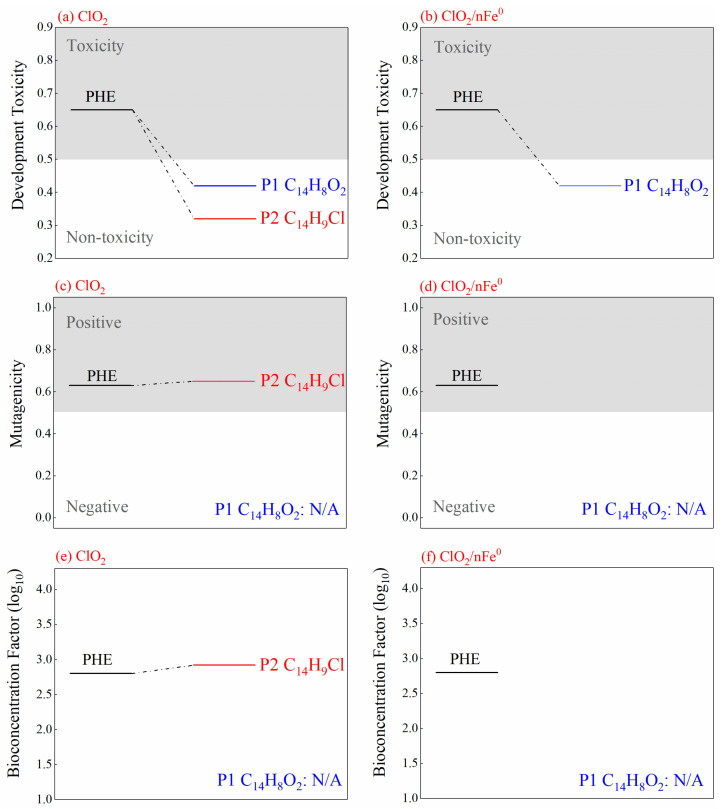
Toxicity estimation of PHE and its degradation products in ClO_2_ system (**a**,**c**,**e**,**g**) and ClO_2_/nFe^0^ system (**b**,**d**,**f**,**h**). N/A means not available.

Tables S5 and S6 list the degradation intermediates of PHE and ANT in the ClO_2_/nFe^0^ system using GC-MS (Figures S5 and S6). The main degradation intermediates of PAHs were chlorinated PAHs and oxygenated PAHs [71]. The main intermediate of PHE was 9,10-phenanthrenedione (P1) in the ClO_2_/nFe^0^ system (Figure 6a). However, P1 and 9-chlorophenanthrene (P2) were generated in the ClO_2_-alone system (Figure 6a and Figure S5). The toxicity estimation software tool was employed to estimate the ecotoxicity of the intermediates [11]. The toxicity of P1 was significantly lower than PHE (Figure 7), while P2 still had high mutagenicity toxicity and bioconcentration factor [72,73]. The results showed that the intermediate of PHE in the ClO_2_/nFe^0^ system had lower toxicity than those in the ClO_2_ system. For the degradation of ANT, anthrone (A1), 9,10-anthracinedione (A2), and benzophenone (A3) were detected in both the ClO_2_/nFe^0^ and ClO_2_-alone systems. However, 4-chlorobenzophenone (A4), which was chlorinated from A3, was presented only in the ClO_2_ system, while anthralin (A5), which takes a dichlorination reaction, existed only in the ClO_2_/nFe^0^ system [74,75]. Toxicity estimation analysis (Figure S7) showed that the bioconcentration factor was 614.34 for ANT, but 14.2 for A5 in the ClO_2_/nFe^0^ system. Above all, the intermediates of the PAHs in the ClO_2_/nFe^0^ system had lower toxicity, indicating a great practical potential of ClO_2_/nFe^0^ for soil treatment.

### 3.5. Lettuce Growth in ClO_2_/nFe^0^-Treated Soil

**Figure 8:**
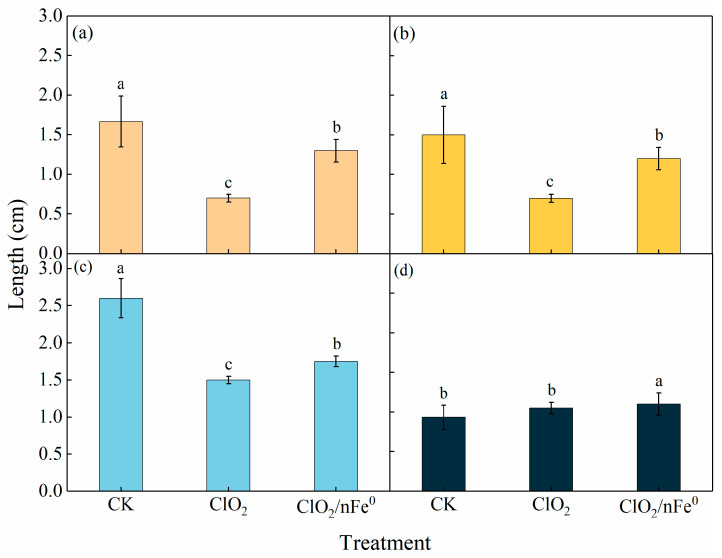
The leaf length and leaf width of lettuce seedings grown in soil treated with ClO_2_/nFe^0^ or nFe^0^-alone systems. The length of the first leaf (**a**), the second leaf (**b**), and the longest leaf (**c**), and the width of the longest leaf (**d**). The different lowercase letters above the bars indicate groups with significant differences (*p* < 0.05).

Figure 8 showed the soil-based bioassays of lettuce in soil treated by ClO_2_/nFe^0^. With 14 d-cultivation, the lettuce plants grown in ClO_2_/nFe^0^-treated soil had longer and wider leaves than those grown in ClO_2_-treated soil, but were smaller than lettuce grown in clean soil. The dry weights of lettuce in different treatments had no significant differences (Figure S8). This could be attributed to the following reasons: the presence of nFe^0^ could act as a necessary nutrient for plants [76]. In addition, the intermediates of PAHs in the ClO_2_/nFe^0^ system had lower toxicity. However, subsequent studies focusing on enhancing the mineralization of PAHs in soil are still worthy of being carried out.

## 4. Conclusions

Herein, a nFe^0^-activated ClO_2_ system was employed to treat PHE-spiked grassland soil. The reaction conditions (the molar ratio of ClO_2_ to nFe^0^, reaction pH, and ratio of water to soil (*w*/*w*)) were optimized to improve the degradation efficiency of PHE. By conducting EPR analysis and quenching experiments, Fe(IV), HClO, and •OH were revealed to be the three most important active oxygen species when ClO_2_ was activated by nFe^0^ with respective oxidation contributions of 34.3%, 52.8%, and 12.9%. The presence of nFe^0^ in the ClO_2_ system decreased the toxicity of degradation intermediates. The lettuces grown in ClO_2_/nFe^0^-treated soil displayed better bioassay indexes than those grown in ClO_2_-treated soil. Overall, these findings support the prospect of remediating organic-pollutant-contaminated soil by using Fe^0^-activated ClO_2_ technology.

## Data Availability

The data that support the findings of this study are available from the corresponding author upon reasonable request.

## Supplementary Materials

The following are available online at https://www.mdpi.com/article/10.3390/toxics13010036/s1: Text S1: Materials and reagents; Text S2: Analytical procedures; Figure S1: The chromatograms of PMSO and PMSO_2_; Figure S2: High-resolution XPS spectra of O 1s for fresh nFe^0^ (a) and used nFe^0^ (b); Figure S3: The concentration of Fe(Ⅲ)_sol_ and Fe(Ⅲ)_surf_ in ClO_2_/nFe^0^ or nFe^0^ system. Experimental conditions: [PHE] = 1 mg/L; [ClO_2_] = 548 mg/L; [nFe^0^] = 100 mg/L; T = 25 °C; pH = 4.5; Figure S4: Quenching experiments and EPR spectrum of (a) ClO^2^ system, (b) ClO_2_/Fe_2_O_3_ system and (c) ClO_2_/FeOOH system under DMPO capture agent. Experimental conditions: [ClO_2_] = 548 mg/L; [Fe_2_O_3_] = 288 mg/L; [FeOOH] = 156 mg/L; [Gly] = 4 mM; [DMPO] = 100 mM; T = 25 °C; reaction time = 10 min; Figure S5: (a)Total ion chromatogram of PHE in different reaction system. The mass spectra of degradation intermediates with retention time at 15.06 min (b) and 17.84 min (c); Figure S6. Total ion chromatogram of ANT in different reaction system (a) and the mass spectra of degradation intermediates with retention time at 17.455 min (b), 17.705 min (c), 13.785 min (d), 15.790 min (e) and 17.515 min (f); Figure S7: Toxicity estimation of ANT and its degradation products in (a,c) ClO_2_ system and (b,d) ClO_2_/nFe^0^ system; Figure S8: Effect of ClO_2_/nFe^0^ system on lettuce dry weight; Table S1: The physicochemical properties of the grassland soil; Table S2: Experimental conditions for each batch degradation experiments; Table S3: pH evolution in different treatment after the reaction; Table S4: PAHs degradation efficiencies comparison of ClO^2^/nFe^0^ system and other published advanced oxidation techniques; Table S5: The characteristic of PHE and degradation products; Table S6 The characteristic of ANT and degradation products; Scheme S1: proposed mechanisms in ClO_2_/nFe^0^ system. References [77,78,79,80] are cited in the Supplementary Materials.
