# Supplementary material for: Activation of ClO2 by Nanoscale Zero-Valent Iron for Efficient Soil Polycyclic Aromatic Hydrocarbon Degradation: New Insight into the Relative Contribution of Fe(IV) and Hydroxyl Radicals
Source: Toxics. 2025 Jan 5;13(1):36. doi: 10.3390/toxics13010036 (PMC11768630; doi:10.3390/toxics13010036)
Supplement: Supplementary file 1 [file toxics-13-00036-s001.zip › toxics-3359634-supplementary.pdf]

Supplementary Information

Activation of ClO_2 by nanoscale zero-valent iron for efficient soil PAHs degradation: new insight into relative contribution of Fe(IV) and hydroxyl radical

Xiaojun Hu[a], Xiaorong Xing[a], Fan Zhang[a], Bingzhi Li[a], Senlin Chen[a], Bo Wang[a], Jiaolong Qin[a]*, Jie Miao[b]*

[a]School of Chemical and Environmental Engineering, Shanghai Institute of Technology, Shanghai, 201418, China

[b]School of Environmental Science and Engineering, Nanjing Tech University, Nanjing 211816, China

*Corresponding author: qinjiaolong@sit.edu.cn (J. Qin); miaojie@njtech.edu.cn (J. Miao)

Texts

Text S1. Materials and reagents

Text S2. Analytical procedures

Figures

Fig.S1. The chromatograms of PMSO and $PMSO_2$.

Fig.S2. High-resolution XPS spectra of O 1s for fresh nFe^0 (a) and used nFe^0 (b).

Fig.S3. The concentration of $Fe(III)_{sol}$ and $Fe(III)_{surf}$ in ClO_2/nFe^0 or nFe^0 system. Experimental conditions: [PHE]=1 mg/L; $[ClO_2]$ = 548 mg/L; $[nFe^0]$ = 100 mg/L; T = 25 ◦C; pH=4.5.

Fig.S4. Quenching experiments and EPR spectrum of (a) ClO_2 system, (b) ClO_2/Fe_2O_3 system and (c) ClO_2/FeOOH system under DMPO capture agent. Experimental conditions: $[ClO_2]$ = 548 mg/L; $[Fe_2O_3]$ =288 mg/L; [FeOOH] =156 mg/L; [Gly] = 4 mM; [DMPO] = 100 mM; T = 25 ◦C; reaction time = 10 min.

Fig.S5. (a)Total ion chromatogram of PHE in different reaction system. The mass spectra of degradation intermediates with retention time at 15.06 min (b) and 17.84 min

(c).

Fig.S6. Total ion chromatogram of ANT in different reaction system (a) and the mass spectra of degradation intermediates with retention time at 17.455 min(b), 17.705 min (c), 13.785 min (d), 15.790 min (e) and 17.515 min (f).

Fig.S7. Toxicity estimation of ANT and its degradation products in (a,c) ClO_2 system and (b, d) ClO_2/nFe^0 system.

Fig.S8. Effect of ClO_2/nFe^0 system on lettuce dry weight.

Tables

Table S1. The physicochemical properties of the grassland soil

Table S2. Experimental conditions for each batch degradation experiments

Table S3. pH evolution in different treatment after the reaction

Table S4. PAHs degradation efficiencies comparison of ClO_2/nFe^0 system and other published advanced oxidation techniques.

Table S5. The characteristic of PHE and degradation products.

Table S6 The characteristic of ANT and degradation products.

Scheme

Scheme S1 proposed mechanisms in ClO_2/nFe^0 system

Experimental

Text S1. Materials and reagents

Chloroform (98%, HPLC), isopropyl alcohol (98%, HPLC) and glycine (99.5%, analytical grade) were purchased from Merck (Darmstadt, Germany), the 5,5-Dimethyl-1-pyrroline-N-oxide (DMPO, 99%, analytical grade) and methyl phenyl sulfoxide (PMSO, 98%, analytical grade) were purchased from Innochem (Beijing, China). Anthracene (ANT, 97%, analytical grade) , phenanthrene (PHE, 99%, analytical grade), sodium chlorite (80%, research grade), haematite (α-Fe_2O_3,99.95%, analytical grade) and goethite (α-FeOOH, 99%, research grade) were obtained from Adamas-beta (Shanghai, China), and Benzo [a] anthracene (98%, analytical grade) from Boer (Shanghai, China). All other chemicals were of analytical grade. The deionized water was ultrasound to remove the dissolve O_2 before used.

Text S2. Analytical procedures

Chromatographic separations were performed using a C18 column (250 mm × 4.6 mm, 5 μm particle size, ODS-3, GL sciences, Shanghai). The UV detector was set as 230 nm for PMSO and 264 nm for $PMSO_2$. The isocratic mobile phase consisted of 50% water and 50% acetonitrile at a flow rate of 0.6 mL/min. The oven temperature was 35 °C, and automatic injection was 20 μL.

Fig.S1

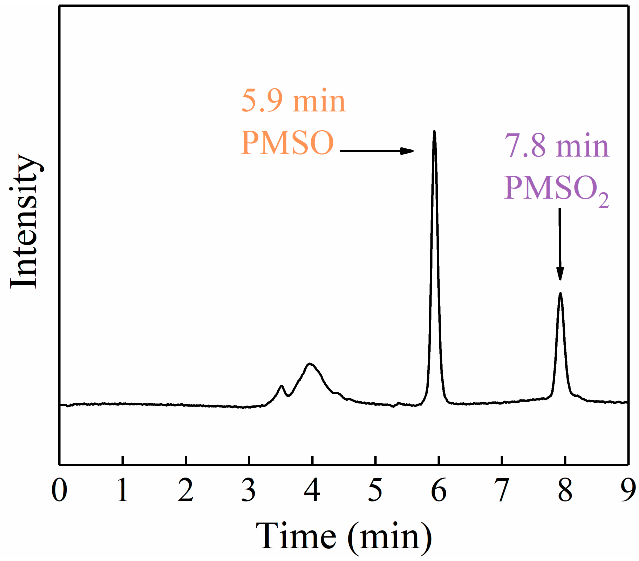

Fig.S2：

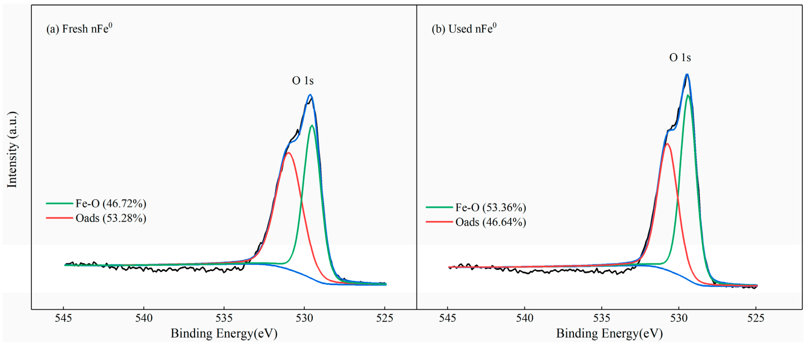
(a) Fresh nFe^0
O 1s
Intensity (a.u.)
Fe-O (46.72%)
Oads (53.28%)
545
540
535
530
525
Binding Energy(eV)
(b) Used nFe^0
O 1s
Fe-O (53.36%)
Oads (46.64%)
545
540
535
530
525
Binding Energy(eV)

Fig.S3.

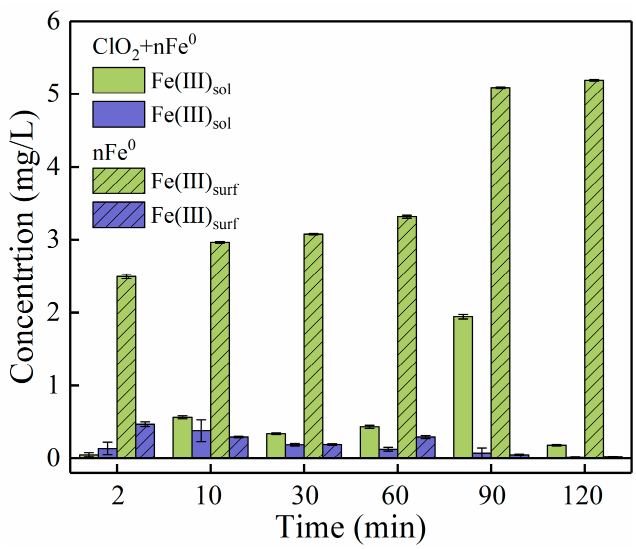
ClO2+nFe0
Fe(III)sol
Fe(III)sol
nFe0
Fe(III)surf
Fe(III)surf
Concentrtion (mg/L)
Time (min)
0
1
2
3
4
5
6
2
10
30
60
90
120

Fig.S4

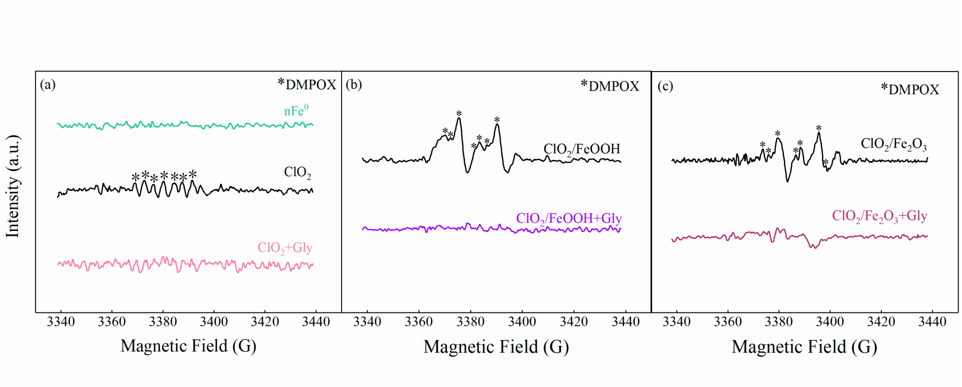
(a)
*DMPOX
nFe^0
ClO$_2$
ClO$_2$+Gly
(b)
*DMPOX
ClO$_2$/FeOOH
ClO$_2$/FeOOH+Gly
(c)
*DMPOX
ClO$_2$/Fe$_2$O$_3$
ClO$_2$/Fe$_2$O$_3$+Gly
Intensity (a.u.)
3340 3360 3380 3400 3420 3440
Magnetic Field (G)

Fig.S5

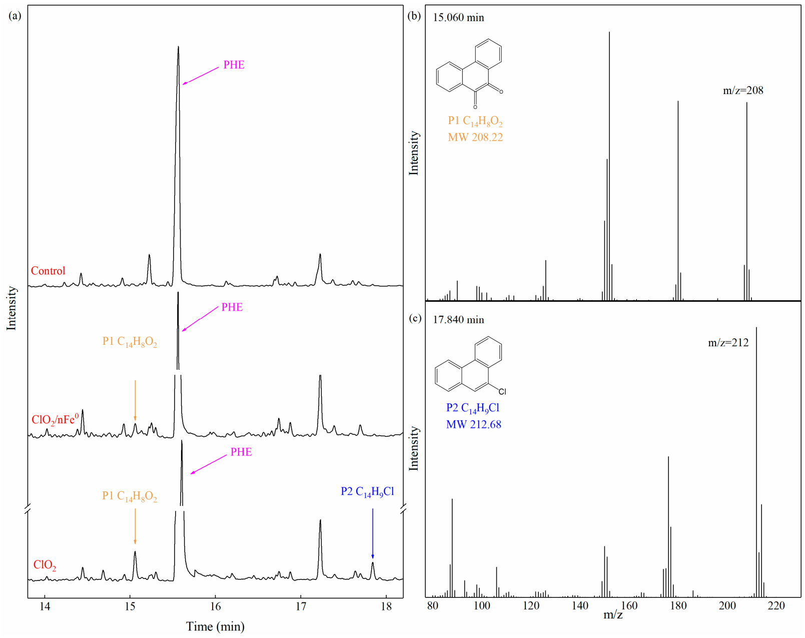
(a)
PHE
Control
PHE
P1 C14H8O2
ClO2/nFe0
PHE
P1 C14H8O2
P2 C14H9Cl
ClO2
Intensity
14
15
16
17
18
Time (min)
(b)
15.060 min
P1 C14H8O2
MW 208.22
m/z=208
Intensity
(c)
17.840 min
Cl
P2 C14H9Cl
MW 212.68
m/z=212
Intensity
80
100
120
140
160
180
200
220
m/z

Fig.S6

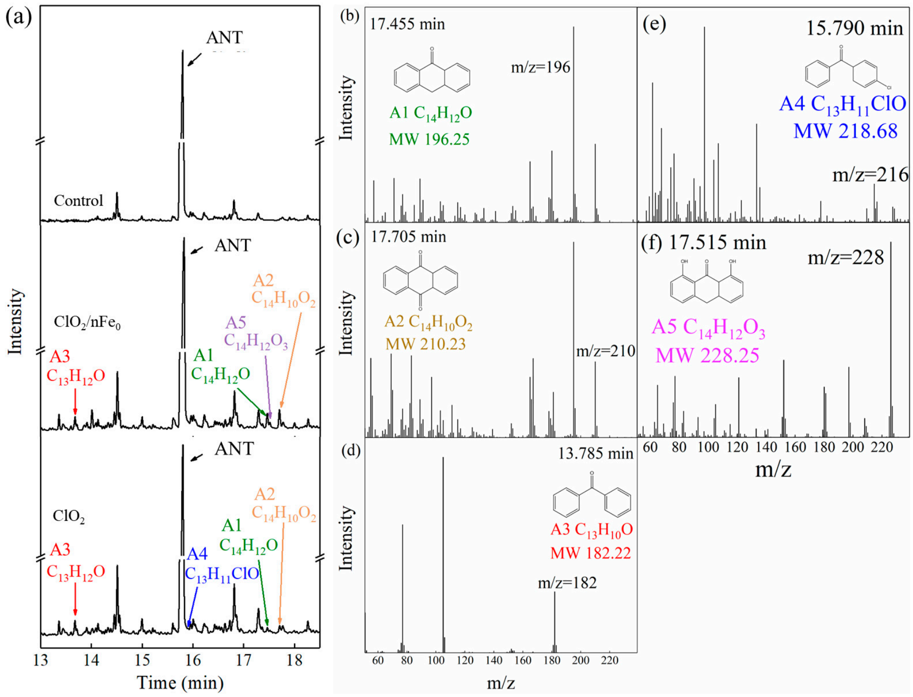
(a)
ANT
Control
ANT
ClO$_2$/nFe$_0$
A2 $C_{14}H_{10}O_2$
A5 $C_{14}H_{12}O_3$
A1 $C_{14}H_{12}O$
A3 $C_{13}H_{12}O$
ANT
ClO$_2$
A2 $C_{14}H_{10}O_2$
A1 $C_{14}H_{12}O$
A4 $C_{13}H_{11}ClO$
A3 $C_{13}H_{12}O$
Intensity
13 14 15 16 17 18
Time (min)
(b) 17.455 min
A1 $C_{14}H_{12}O$
MW 196.25
m/z=196
(c) 17.705 min
A2 $C_{14}H_{10}O_2$
MW 210.23
m/z=210
(d) 13.785 min
A3 $C_{13}H_{10}O$
MW 182.22
m/z=182
(e) 15.790 min
A4 $C_{13}H_{11}ClO$
MW 218.68
m/z=216
(f) 17.515 min
m/z=228
A5 $C_{14}H_{12}O_3$
MW 228.25
60 80 100 120 140 160 180 200 220
m/z

Fig.S7

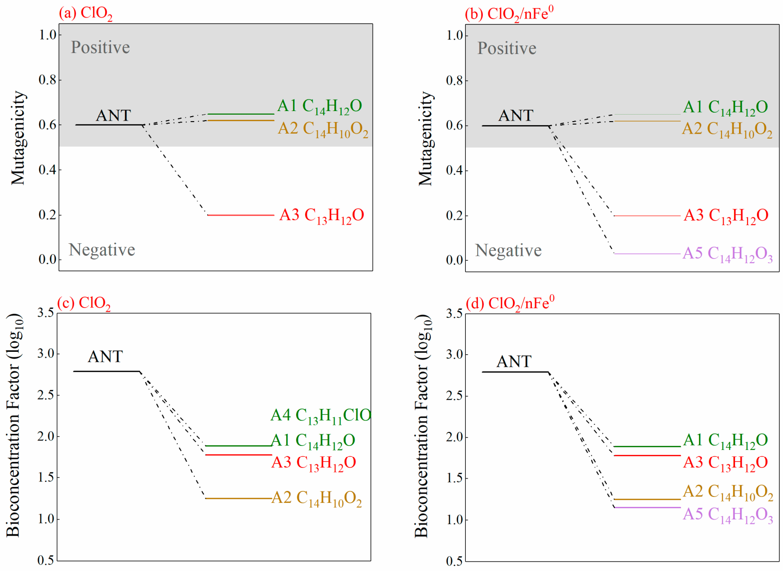
(a) ClO$_2$
Positive
Negative
Mutagenicity
ANT
A1 C$_{14}$H$_{12}$O
A2 C$_{14}$H$_{10}$O$_2$
A3 C$_{13}$H$_{12}$O
(b) ClO$_2$/nFe^0
A5 C$_{14}$H$_{12}$O$_3$
(c) ClO$_2$
Bioconcentration Factor (log$_{10}$)
A4 C$_{13}$H$_{11}$ClO
(d) ClO$_2$/nFe^0

Fig.S8

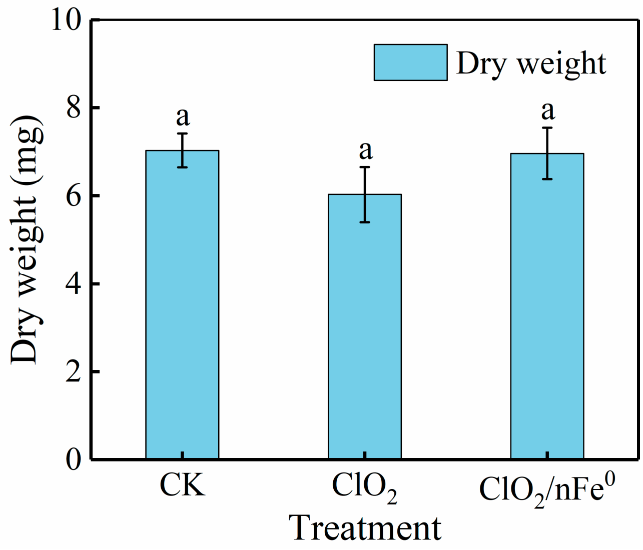

Dry weight
Dry weight (mg)
10
8
6
4
2
0
a
a
a
CK
ClO2
ClO2/nFe0
Treatment

Table S1.

Parameters	Value	Unit
Silt	79.20	wt%
Clay	14.90	wt%
Sand	5.90	wt%
Soil organic matter (SOM)	2.00±0.00	wt%
Moisture content	25.10±0.80	%
Cation exchange capacity	11.70±0.40	cmol/kg
pH (in water)	7.10±0.00	/
Total iron (Fe)	550.13±1.76	mg/kg

Table S2

Variables	Operation conditions
Water-soil ratios<br>(1:1~5:1)	ClO_2 = 80 mM/kg<br>nFe^0 = 0.33 g/kg<br>pH = 4.5 (without adjustment)
ClO_2 Dosage<br>(40、80、160 mM/kg)	Water-soil ratio = 3:1<br>nFe^0 = 0.33 g/kg<br>pH = 4.5 (without adjustment)
nFe^0 Dosage<br>(0.25、0.33、0.5、1、2.5 g/kg)	Water-soil ratio = 3:1<br>ClO_2 = 80 mM/kg<br>pH = 4.5 (without adjustment)
pH<br>(3.0、4.0、4.5、6.0、9.0)	Water-soil ratio = 3:1<br>ClO_2 = 80 mM/kg<br>nFe^0 = 0.33 g/kg

Table S3

Operation conditions	Initial pH	Final pH
Water-soil ratio = 3:1<br>ClO_2 = 80 mM/kg<br>nFe^0 = 0.33 g/kg	3.0	5.9±0.5
	4.0	5.9±0.2
	4.5	5.6±0.3
	6.0	7.3±0.3
	9.0	7.2±0.3

Table S4

Technique	Oxidant (mg/kg soil)	Oxidant/pollutant (mg/mg)	Operation conditions	Efficiency	Ref.
Soil's natural iron/ persulfate	Persulfate = 10 g/kg	Persulfate/PHE=1.7	PHE = 6000 mg/kg<br>pH = 7<br>T = room temperature<br>t = 24 h	81 %	[77]
Sodium percarbonate / H_2O_2	H_2O_2 = 158.5 g/kg	H_2O_2/PAHs=30	PHE = 5116 mg/kg<br>T = 40℃<br>t = 6 h	53%	[78]
H_2O_2	H_2O_2 = 38.5g/kg	H_2O_2/PAHs=24.8	PAHs =1550 mg/kg<br>T = room temperature<br>t = 9 h	45%	[36]
CMC-Fe_3O_4@BC/ H_2O_2	H_2O_2 = 17 g/kg	H_2O_2/PAHs=383.7	PAHs = 44.3 mg/kg<br>T = room temperature<br>t = 10 d	66.1%	[79]
Fe^{2+}/oxalic acid /calcium peroxide /persulfate	Persulfate = 42.8 g/kg<br>Calcium peroxide = 8.89 g/kg	Oxidant/ PAHs=375.1	PAHs = 137.8 mg/kg<br>T = room temperature	70.8%	[80]

ClO_2/nFe^0	ClO_2=1.6 g/kg	ClO_2/PHE=16.4	PHE = 100 mg/kg<br>pH = 4.5<br>T = room temperature<br>t= 12 h	57.9%	This study

[77] Qutob, M., Rafatullah, M., Muhammad, S.A., Siddiqui, M.R., Alam, M., 2024. Advanced oxidation of polycyclic aromatic hydrocarbons in tropical soil: Self-catalytic utilization of natural iron contents in an oxygenation reactor supported with persulfate. Sci. Total Environ. 926, 171843. https://doi.org/https://doi.org/10.1016/j.scitotenv.2024.171843.

[78] Cajal-Mariñosa, P., Calle, R.G.d.l., Rivas, F.J., Tuhkanen, T., 2012. Impacts of Changing Operational Parameters of In Situ Chemical Oxidation (ISCO) on Removal of Aged PAHs from Soil. Journal of Advanced Oxidation Technologies 15(2). https://doi.org/10.1515/jaots-2012-0223.

[79] Gao, Y., Xue, Y., Zhen, K., Guo, J., Tang, X., Zhang, P., Wang, C., Sun, H., Wu, J., 2023. Remediation of soil contaminated with PAHs and γ-HCH using Fenton oxidation activated by carboxymethyl cellulose-modified iron oxide-biochar. J Hazard Mater 453, 131450. https://doi.org/https://doi.org/10.1016/j.jhazmat.2023.131450.

[80] Wang, J., Zhang, X., Zhou, X., Waigi, M.G., Gudda, F.O., Zhang, C., Ling, W., 2021. Promoted oxidation of polycyclic aromatic hydrocarbons in soils by dual persulfate/calcium peroxide system. Sci. Total Environ. 758. https://doi.org/10.1016/j.scitotenv.2020.143680.

Table S5

No.	Chemical name	Chemical formula	Retention time/min	Molecular structure	Detected in
	Phenanthrene	$C_{14}H_{10}$	15.595	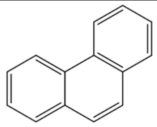	ClO_2/nFe^0<br>ClO_2
P1	9,10-Phenanthrenedione	$C_{14}H_8O_2$	15.060	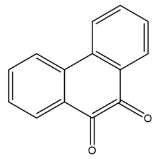	ClO_2/nFe^0<br>ClO_2
P2	9-Chlorophenanthrene	$C_{14}H_9Cl$	17.840	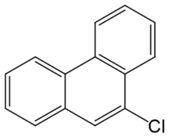	ClO_2

Table S6

No.	Chemical name	Chemical formula	Retention time/min	Molecular structure	Detected in
	Anthracene	$C_{14}H_{10}$	15.74	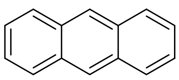	ClO_2/nFe^0<br>ClO_2
A1	Anthrone	$C_{14}H_{12}O$	17.45	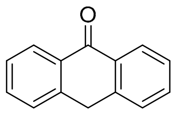	ClO_2/nFe^0<br>ClO_2
A2	9,10-Anthracenedione	$C_{14}H_{10}O_2$	17.70	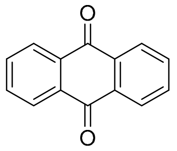	ClO_2/nFe^0<br>ClO_2
A3	Benzophenone	$C_{13}H_{12}O$	13.78	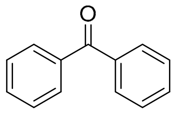	ClO_2/nFe^0<br>ClO_2
A4	4-Chlorobenzophenone	$C_{13}H_{11}ClO$	15.79	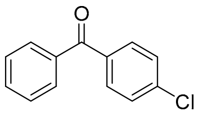	ClO_2
A5	Anthralin	$C_{14}H_{12}O_3$	17.52	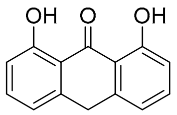	ClO_2/nFe^0

Scheme S1

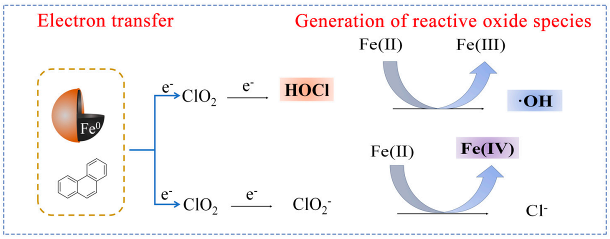

Electron transfer
Generation of reactive oxide species
Fe(II)
Fe(III)
Fe(II)
Fe(IV)
Fe0
e-
ClO2
e-
HOCl
·OH
e-
ClO2
e-
ClO2-
Cl-
